# Association of Lifecourse Socioeconomic Status with Chronic Inflammation and Type 2 Diabetes Risk: The Whitehall II Prospective Cohort Study

**DOI:** 10.1371/journal.pmed.1001479

**Published:** 2013-07-02

**Authors:** Silvia Stringhini, G. David Batty, Pascal Bovet, Martin J. Shipley, Michael G. Marmot, Meena Kumari, Adam G. Tabak, Mika Kivimäki

**Affiliations:** 1 Institute of Social and Preventive Medicine (IUMSP), Lausanne University Hospital, Lausanne, Switzerland; 2 University College London, Department of Epidemiology and Public Health, London, United Kingdom; 3 Centre for Cognitive Ageing and Cognitive Epidemiology, University of Edinburgh, Edinburgh, United Kingdom; 4 1st Department of Medicine, Semmelweis University Faculty of Medicine, Budapest, Hungary; Chinese University of Hong Kong, China

## Abstract

**Background:**

Socioeconomic adversity in early life has been hypothesized to “program” a vulnerable phenotype with exaggerated inflammatory responses, so increasing the risk of developing type 2 diabetes in adulthood. The aim of this study is to test this hypothesis by assessing the extent to which the association between lifecourse socioeconomic status and type 2 diabetes incidence is explained by chronic inflammation.

**Methods and Findings:**

We use data from the British Whitehall II study, a prospective occupational cohort of adults established in 1985. The inflammatory markers C-reactive protein and interleukin-6 were measured repeatedly and type 2 diabetes incidence (new cases) was monitored over an 18-year follow-up (from 1991–1993 until 2007–2009). Our analytical sample consisted of 6,387 non-diabetic participants (1,818 women), of whom 731 (207 women) developed type 2 diabetes over the follow-up. Cumulative exposure to low socioeconomic status from childhood to middle age was associated with an increased risk of developing type 2 diabetes in adulthood (hazard ratio [HR] = 1.96, 95% confidence interval: 1.48–2.58 for low cumulative lifecourse socioeconomic score and HR = 1.55, 95% confidence interval: 1.26–1.91 for low-low socioeconomic trajectory). 25% of the excess risk associated with cumulative socioeconomic adversity across the lifecourse and 32% of the excess risk associated with low-low socioeconomic trajectory was attributable to chronically elevated inflammation (95% confidence intervals 16%–58%).

**Conclusions:**

In the present study, chronic inflammation explained a substantial part of the association between lifecourse socioeconomic disadvantage and type 2 diabetes. Further studies should be performed to confirm these findings in population-based samples, as the Whitehall II cohort is not representative of the general population, and to examine the extent to which social inequalities attributable to chronic inflammation are reversible.

*Please see later in the article for the Editors' Summary*

## Abstract 2

Silvia Stringhini and colleagues followed a group of British civil servants over 18 years to look for links between socioeconomic status and health.

*Please see later in the article for the Editors' Summary*

## Editors' Summary

**Background:**

Worldwide, more than 350 million people have diabetes, a metabolic disorder characterized by high amounts of glucose (sugar) in the blood. Blood sugar levels are normally controlled by insulin, a hormone released by the pancreas after meals (digestion of food produces glucose). In people with type 2 diabetes (the commonest form of diabetes) blood sugar control fails because the fat and muscle cells that normally respond to insulin by removing sugar from the blood become insulin resistant. Type 2 diabetes, which was previously called adult-onset diabetes, can be controlled with diet and exercise, and with drugs that help the pancreas make more insulin or that make cells more sensitive to insulin. However, as the disease progresses, the pancreatic beta cells, which make insulin, become impaired and patients may eventually need insulin injections. Long-term complications, which include an increased risk of heart disease and stroke, reduce the life expectancy of people with diabetes by about 10 years compared to people without diabetes.

**Why Was This Study Done?:**

Socioeconomic adversity in childhood seems to increase the risk of developing type 2 diabetes but why? One possibility is that chronic inflammation mediates the association between socioeconomic adversity and type 2 diabetes. Inflammation, which is the body's normal response to injury and disease, affects insulin signaling and increases beta-cell death, and markers of inflammation such as raised blood levels of C-reactive protein and interleukin 6 are associated with future diabetes risk. Notably, socioeconomic adversity in early life leads to exaggerated inflammatory responses later in life and people exposed to social adversity in adulthood show greater levels of inflammation than people with a higher socioeconomic status. In this prospective cohort study (an investigation that records the baseline characteristics of a group of people and then follows them to see who develops specific conditions), the researchers test the hypothesis that chronically increased inflammatory activity in individuals exposed to socioeconomic adversity over their lifetime may partly mediate the association between socioeconomic status over the lifecourse and future type 2 diabetes risk.

**What Did the Researchers Do and Find?:**

To assess the extent to which chronic inflammation explains the association between lifecourse socioeconomic status and type 2 diabetes incidence (new cases), the researchers used data from the Whitehall II study, a prospective occupational cohort study initiated in 1985 to investigate the mechanisms underlying previously observed socioeconomic inequalities in disease. Whitehall II enrolled more than 10,000 London-based government employees ranging from clerical/support staff to administrative officials and monitored inflammatory marker levels and type 2 diabetes incidence in the study participants from 1991–1993 until 2007–2009. Of 6,387 participants who were not diabetic in 1991–1993, 731 developed diabetes during the 18-year follow-up. Compared to participants with the highest cumulative lifecourse socioeconomic score (calculated using information on father's occupational position and the participant's educational attainment and occupational position), participants with the lowest score had almost double the risk of developing diabetes during follow-up. Low lifetime socioeconomic status trajectories (being socially downwardly mobile or starting and ending with a low socioeconomic status) were also associated with an increased risk of developing diabetes in adulthood. A quarter of the excess risk associated with cumulative socioeconomic adversity and nearly a third of the excess risk associated with low socioeconomic trajectory was attributable to chronically increased inflammation.

**What Do These Findings Mean?:**

These findings show a robust association between adverse socioeconomic circumstances over the lifecourse of the Whitehall II study participants and the risk of type 2 diabetes and suggest that chronic inflammation explains up to a third of this association. The accuracy of these findings may be affected by the measures of socioeconomic status used in the study. Moreover, because the study participants were from an occupational cohort, these findings need to be confirmed in a general population. Studies are also needed to examine the extent to which social inequalities in diabetes risk that are attributable to chronic inflammation are reversible. Importantly, if future studies confirm and extend the findings reported here, it might be possible to reduce the social inequalities in type 2 diabetes by promoting interventions designed to reduce inflammation, including weight management, physical activity, and smoking cessation programs and the use of anti-inflammatory drugs, among socially disadvantaged groups.

**Additional Information:**

Please access these Web sites via the online version of this summary at http://dx.doi.org/10.1371/journal.pmed.1001479.

- The US National Diabetes Information Clearinghouse provides information about diabetes for patients, health-care professionals, and the general public, including information on diabetes prevention (in English and Spanish)
- The UK National Health Service Choices website provides information for patients and carers about type 2 diabetes; it includes peoples stories about diabetes
- The nonprofit Diabetes UK also provides detailed information about diabetes for patients and carers, including information on healthy lifestyles for people with diabetes, and has a further selection of stories from people with diabetes; the nonprofit Healthtalkonline has interviews with people about their experiences of diabetes
- MedlinePlus provides links to further resources and advice about diabetes (in English and Spanish)
- Information about the Whitehall II study is available

## Introduction

A large body of evidence suggests that socioeconomically disadvantaged groups experience an increased risk of type 2 diabetes [1],[2], a metabolic disorder characterized by chronic hyperglycemia, insulin resistance, and impaired beta-cell function [3]. Early life factors are thought to be implicated in the development of type 2 diabetes [4]–[6]. In particular, in observational studies, social adversity in childhood has been related to an increased incidence of adult type 2 diabetes [7],[8] and its risk factors, such as the metabolic syndrome [9],[10], elevated insulin resistance [11], and raised blood glucose [12]. Type 2 diabetes is an increasingly common chronic condition [13],[14], as well as being an important risk factor for premature mortality, cardiovascular disease, and depression [15]–[17]. A better understanding of the mechanisms involved in the socioeconomic distribution of type 2 diabetes is therefore essential for tackling social inequalities in this disorder.

Traditionally, the mechanisms that have been proposed to explain the apparent “long-reach” of early-life socioeconomic circumstances on type 2 diabetes risk include mediation by diabetes risk factors such as obesity, physical inactivity, and diet [8],[18],[19]. More recently, adverse socioeconomic circumstances have also been suggested to be associated with up-regulation of genes affecting white blood cell count and down-regulation of genes controlling immune cells responsiveness to glucocorticoid signaling [20]. Evidence is also accumulating for a more fundamental role of social and financial adversities over the entire lifespan in programming a “vulnerable” phenotype that, through glucocorticoid receptor resistance, leads to exaggerated glucocorticoid levels and exacerbated inflammatory responses in adult life [12],[20]–[24].

The effect of social adversity on inflammation-related gene regulation might not be limited to early life experiences, however. An experimental study in fully grown macaques, for example, found that changes in the social environment in mid-life affected the expression of genes regulating the immune system, contributing to an elevated inflammatory response [25]. This finding is in agreement with studies on humans, showing greater inflammation in people exposed to social adversity especially in adulthood [26]–[30]. In addition, low socioeconomic status (SES) across the lifecourse has been consistently shown to predict the risk of inflammation-related chronic conditions, such as cardiovascular disease (CVD) and type 2 diabetes [1],[2],[7],[11],[31].

Biologically, chronic inflammation is a plausible mediator of the association between socioeconomic adversity and type 2 diabetes. Inflammation affects insulin signalling [32] and increases beta-cell death [33], and markers of inflammation, such as elevated interleukin 6 (IL-6) and C-reactive protein (CRP) levels, have been found to be associated with future diabetes risk [34],[35]. Inflammation may also increase type 2 diabetes risk indirectly via obesity, which, as described, is a risk factor for type 2 diabetes and is associated with increased release of inflammatory markers, such as IL-6 [36].

Taking together the evidence linking socioeconomic adversity to inflammation and inflammation to type 2 diabetes, it seems reasonable to postulate that chronically increased inflammatory activity in individuals exposed to socioeconomic adversity over the entire lifecourse may, at least partially, mediate the association between SES over the lifecourse and future type 2 diabetes risk. In order to test this hypothesis, we first explore the association between lifecourse SES and type 2 diabetes incidence, and then examine the extent to which this association is explained, if at all, by inflammatory markers.

## Materials and Methods

### Study Population and Design

Established in 1967, the focus of the original Whitehall study was to understand the aetiology of CVD. One of the major findings from the study was the lower rates of CVD mortality in the highest employment grade groups [37]. This observation led to the initiation of the Whitehall II study in 1985 to investigate a range of possible mechanisms potentially underlying these socioeconomic inequalities in disease, most notably psychosocial stress. The Whitehall II study has now matured to the extent that it comprises multiple follow-up screenings and questionnaire surveys [38]. Thus, very unusually and of particular relevance to the present analyses, this study has repeat measures of systemic inflammation and type 2 diabetes incidence over the adult lifecourse.

The Whitehall II study comprised 10,308 (3,413 women) London-based civil servants (government employees) aged 35–55 y at study induction [38]. The first examination (phase 1) took place during 1985–1988, and involved a clinical examination and a self-administered questionnaire. A 75 g oral glucose tolerance test (OGTT) was performed for the first time at phase 3 (1991–1993; *n* = 8,815) and repeated at phase 5 (1997–1999), phase 7 (2003–2004), and phase 9 (2007–2009). Therefore, phase 3 examination is the “baseline” for the present analyses. Participants free of type 2 diabetes at phase 3 were included and followed for incident diabetes up to phase 9, a total of 18 y. Additional questionnaire-only phases also assessed diabetes status at phase 4 (1995–1996), phase 6 (2001), and phase 8 (2006). The study was approved by the University College London ethics committee, and all participants provided written consent.

### Lifecourse Socioeconomic Status

Three indicators of SES over the lifecourse were used: father's occupational position, the study member's educational attainment, and adult occupational position. These three indicators were selected to cover the study members' lifespan. Father's occupational position is a common indicator of SES in childhood in the United Kingdom [39]. Education is also a measure of SES in early life but, being generally acquired in adolescence or young adulthood, it can be considered as a measure of SES before active professional life [40],[41]. Finally, adult occupational position is one of the most used indicators of adult SES [42].

Father's occupational position was assessed retrospectively at the baseline survey (phase 1) with the question “What is/was your father's main job, what kind of work does/did he do in it.” This was coded based on the Registrar General's Occupational position classification [43] and then categorized as high (social classes I–II), middle (social classes III NM–III M), and low (social classes IV–V). For 310 participants, missing data on father's occupational position were replaced with data on father's education, categorized as high (≥16 y of schooling), middle (14–16 y of schooling), and low (<14 y of schooling).

Education was drawn from phase 5 of the study (1997–1999) and it was assessed as the highest qualification attained while in full-time education. It was grouped into three categories: high (university degree), middle (higher secondary school), and low (lower than higher secondary school). For non-responders at phase 5 (*n* = 2,377), the baseline (phase 1) measure of education was used.

Adult occupational position was based on the employment grade at phase 3 and categorized into high (administrative), middle (professional/executive), and low (clerical/support).

At least three conceptual models describe the impact of lifecourse socioeconomic circumstances on health in adulthood: (1) latent effects of early life socioeconomic circumstances on adult health; (2) cumulative effect of exposure to adverse socioeconomic circumstances from across the lifecourse that affect health in a dose-response manner; and (3) pathways effects of early life socioeconomic circumstances on individuals' trajectories to SES in adulthood, that in turn has an impact on health [44],[45]. To address all the conceptual models, we compare different indicators of SES over the lifecourse for their effect on type 2 diabetes and also assess the impact of cumulative exposure to low SES across the lifecourse and downward lifecourse socioeconomic trajectories on adult onset of type 2 diabetes.

A cumulative SES score was calculated using information on father's occupational position, participants' education, and participants' occupational position at phase 3. Each SES measure was a 3-level variable with values ranging from 0 (high) to 2 (low). A score was calculated by summing all SES measures (range 0–6). The final cumulative SES score was further categorized as high (score = 0–2, *n* = 3,008), moderate (score = 3–5, *n* = 3,212), and low (score = 6, n = 167).

Socioeconomic trajectories from childhood to adulthood were calculated using information on the father's occupational position and the study member's occupational position. For the purposes of deriving this variable, father's occupational position was dichotomized as high (social classes I-II-III NM) and low (social classes III M-IV-V). Occupational position of the participants was categorized as high (administrative) or low (professional/executive and clerical/support). Four trajectories were therefore possible: high SES in childhood and high SES in adulthood (high-high, *n* = 1,805), low SES in childhood and high SES in adulthood (low-high, *n* = 772), high SES in childhood and low SES in adulthood (high-low, *n* = 2,038), and low SES in childhood and low SES in adulthood (low-low, *n* = 1,772).

All SES indicators were significantly correlated, with Spearman's correlation coefficients ranging between 0.18 and 0.84 (Table S1).

### Incident Type 2 Diabetes

At study phases 3, 5, 7, and 9, venous blood was taken after a ≥5-h fast before consenting participants underwent a standard 75 g 2-h OGTT. Glucose samples were drawn into fluoride Monovette tubes and centrifuged on site within one hour. Blood glucose was measured using the glucose oxidase method, as previously described [46]. At each phase, diabetes was defined by the World Health Organization (WHO) criteria based on fasting glucose ≥7.0 mmol/l or 2-h glucose ≥11.1 mmol/l [3]. Participants reporting doctor diagnosed diabetes or use of diabetes medication were classified as having diabetes regardless of their OGTT results. The date of diabetes diagnosis was assigned according to the interval method as the midpoint between the first visit with a diabetes diagnosis and the last visit without diabetes [46].

### Inflammatory Markers

Fasting serum was collected between 8 am and 1 pm at phases 3, 5, and 7 and stored at −70°C until analysis. CRP was measured using a high-sensitivity immunonephelometric assay in a BN ProSpec nephelometer (Dade Behring) [47]. IL-6 was measured using a high-sensitivity ELISA assay (R & D Systems). Values lower than the detection limit (0.154 mg/l for CRP and 0.08 pg/ml for IL-6) were assigned a value equal to half the detection limit. For CRP at phases 3, 5, and 7 there are 487 (6.4%), 287 (4.6%), and 116 (1.9%) study members, respectively, who had their value set at 0.077. For IL-6, there were no values below the detection limit at any of the three phases. To examine short-term biological variation and laboratory error, a repeated sample was taken from a subset of 150 participants for CRP and 241 for IL-6 at phase 3 (average elapse time between samples was 32 [standard deviation (SD) = 10.5] d), and of 533 for CRP and 329 for IL-6 at phase 7 (average elapse time was 24 [SD = 11.0] d). Reliability between samples was assessed with Pearson's correlation coefficients: r = 0.77 for CRP and r = 0.61 for IL-6 at phase 3 and r = 0.72 for CRP and r = 0.63 for IL-6 at phase 7 [48]. In all analyses, CRP and IL-6 were log-transformed.

### Covariates

Current smoking was self-reported at phases 1, 3, 5, and 7, and classified as yes/no. Physical activity was assessed by using questions on the frequency and duration of participation in moderate or vigorous physical activity at phases 1 and 3. At phases 5 and 7, the questionnaire included 20 items on frequency and duration of participation in different physical activities that were used to calculate hours per week at each intensity level [49]. Participants were classified as “active” (≥2.5 h/wk of moderate or ≥1 h/wk of vigorous physical activity), “inactive” (≤1 h/wk of moderate and ≤1 h/wk of vigorous physical activity), or “moderately active” (if not active or inactive).

Overall diet was assessed by computing a score of adherence to healthy dietary guidelines provided by the Alternative Healthy Eating Index (AHEI) [50],[51]. The AHEI was based on intake levels of vegetables, fruit, nuts and soy, white-to-red meat ratio, total fiber, trans fat, polyunsaturated-to-saturated fatty acids ratio, long-term multivitamin use, and alcohol consumption [51]. The score was then trichotomized based on tertiles. As the AHEI was not available for phase 1, a diet score was computed using information on fruit and vegetable intake and the type of bread and milk most commonly consumed, as described previously [52]. Total carbohydrates intake (measured in grams per day and then categorized in tertiles) was separately included in the analyses as an additional component of diet.

Height and weight were measured directly at phases 1, 3, 5, and 7 using standard procedures. Body mass index (BMI) was then calculated as weight in kilograms divided by height in meters squared, and categorized in three groups (normal <25; overweight 25–29; obese ≥30 kg/m^2^) on the basis of the World Health Organization (WHO) recommendation [53].

Classification of ethnic group was by observer; at the phase 1 screening a study team member classified participants as white Caucasian, South Asian, Afro–Caribbean, Chinese, other or uncertain. Ethnicity was further classified as white/non-white for this study. Family history of type 2 diabetes (parents and siblings) was self-reported at phases 1 and 2 and was categorized as yes/no. Prevalent conditions considered were prevalent coronary heart disease, prevalent stroke, prevalent cancer, and prevalent hypertension (systolic/diastolic blood pressure greater or equal to140/90 mmHg). Age at phase 3 and sex were considered as covariates in the analyses.

### Statistical Analysis

A complete case approach in proportional hazards regression models has been shown to be problematic when data are not missing at random [54]. To reduce this bias, we used an imputation procedure to replace missing values on health behaviours and inflammatory markers. Missing values on smoking, physical activity, diet, and BMI were replaced using information collected at the previous or the successive phases (5 to 10 y earlier or after) (see Table S2) [52]. Missing values on inflammatory markers were imputed using multivariate imputation based on sex, age, ethnicity, BMI, health behaviours, and, for phases 5 and 7, also on inflammatory markers at the preceding phase (Table S2). Missing values on main exposure and outcome variables (socioeconomic indicators, diabetes status), and family history of diabetes were not imputed. Sensitivity analyses repeated on the non-imputed subsample yielded largely similar results (Table S3).

The association between indicators of SES across the lifecourse, the cumulative SES score, and lifecourse SES trajectories with health behaviours, obesity, and high levels of inflammatory markers was assessed using logistic regressions adjusted for age, sex, ethnicity, family history of diabetes, and prevalent conditions. We examined the association of smoking, physical activity, diet, BMI, and inflammatory markers assessed at phase 3 with incident type 2 diabetes using Cox regressions with time-to-event as the time-scale [55]. Cox regressions were also used to examine the association between lifecourse SES and type 2 diabetes. First, father's occupational position, education, and adult occupational position were entered individually into the Cox regression models. Second, we assessed the association between our cumulative SES score and future type 2 diabetes. As tests did not suggest departure from a linear trend (*p* for departure for a linear trend ≥0.05), the cumulative SES score was assessed as a continuous 3-level variable. The hazard ratio (HR) associated with a unit change in SES was squared to yield the HR in the lowest versus the highest cumulative SES category. Third, we examined the association of SES trajectories from childhood to adulthood with type 2 diabetes incidence.

Cox regression models used to assess the SES-type 2 diabetes incidence association were first adjusted for age, sex, ethnicity, family history of diabetes, and prevalent conditions (model 1). Then, smoking, physical activity, diet, BMI, and inflammatory markers were entered first individually and then simultaneously into model 1. The contribution of each risk factor in explaining the SES-type 2 diabetes association was determined by the percent attenuation in the β coefficient for SES after inclusion of the risk factor in question to model 1: “100×(β_Model 1_−β_Model 1+risk factor(s)_)/(β_Model 1_)”.We calculated a 95% CI around the percentage attenuation using a bootstrap method with 1,000 re-samplings.

In analyses of the contribution of mediating factors to the association between SES indicators and type 2 diabetes incidence, we entered smoking, physical activity, diet, BMI, and inflammatory markers in the Cox regression models as time-dependent covariates updated at phases 3, 5, and 7. This procedure allows for changes in the values of the covariates over the follow-up for type 2 diabetes to be taken into account. Further, to account for long-term exposure to these risk factors, at each follow-up period we controlled for the risk factors at the previous phase. Thus, for the follow-up period between phases 3 and 5, risk factors assessed at phase 3 were entered into the model together with the risk factors assessed at phase 1 (except for inflammatory markers that were not measured at phase 1). For the diabetes follow-up period between phases 5 and 7, risk factors collected at phases 3 and 5 were entered simultaneously, and for the follow-up period between phases 7 and 9, risk factors from phases 5 and 7 were entered together. As our study assesses type 2 diabetes incidence, for each follow-up period inflammatory markers are assessed premorbidly (i.e., between 5 and 10 y before the occurrence of diabetes), thus limiting the reciprocal confounding between type 2 diabetes and inflammatory activity (see Figure S1). The proportional hazard assumptions of the Cox regression models were tested using Schoenfeld residuals and found not to be violated (all *p*-values≥0.05). The analyses were performed using the statistical software STATA 12.1, StataCorp LP and SAS 9.2, SAS Institute Inc (%BOOT and %BOOTCI macros).

## Results

Of the 8,815 participants who took part in the Whitehall II phase 3 examination, 2,429 were excluded due to one or more of the following reasons: prevalent type 2 diabetes at phase 3 (*n* = 162), missing follow-up on type 2 diabetes status (*n* = 588), serum CRP levels >10 mg/l at phase 3 or 5 (*n* = 356) indicating acute infection, missing data on SES indicators, such as father's occupational position (*n* = 754) or education (*n* = 696), missing data on inflammatory markers CRP (*n* = 721) or serum IL-6 (*n* = 723) at phase 3 (**Table S2**). Excluded participants had a somewhat lower socioeconomic profile than included participants (27% of excluded versus 14% of the included participants were in the lowest occupational group, *p*<0.001). There were no differences in type 2 diabetes incidence between the included and excluded sample (*p* = 0.147) (Table S4). During the mean 14.3 y follow-up, 731 incident type 2 diabetes cases were identified: 52% on the basis of 75 g OGTT, 23% by use of diabetes medication, and 25% by physician diagnosis.

Our analytical sample consisted of 6,387 participants (1,818 women). Table 1 shows baseline characteristics of the participants according to indicators of SES across the lifecourse. Participants with a low SES were older than those with a high SES (*p*<0.001). The prevalence of family history of diabetes, of prevalent conditions, and of type 2 diabetes incidence were also higher among participants with low versus high adult occupational position (*p*<0.001).

**Table 1:** Study participant characteristics at baseline (Whitehall II phase 3) and type 2 diabetes incidence at a mean 14.3-y follow-up (from phase 3 to phase 9) according to indicators of socioeconomic status in early and adult life (*n* = 6,387; 731 incident diabetes cases).

Characteristics	Father's Occupational Position				Education				Adult Occupational Position			
	High	Medium	Low	*p* a	High	Medium	Low	*p* a	High	Medium	Low	*p* a
*n* (%)	2,803 (43.9)	2,655 (41.6)	929 (14.5)		1,863 (29.2)	1,661 (26.0)	2,863 (44.8)		2,577 (40.3)	2,905 (45.5)	905 (14.2)	
Age, mean (SD)	48.7 (5.9)	49.7 (6.0)	50.0 (6.1)	*<0.001*	47.9 (5.8)	48.6 (5.7)	50.8 (6.1)	*<0.001*	49.5 (5.8)	48.6 (6.1)	51.1 (6.12)	*<0.001*
Men, *n* (%)	2070 (73.9)	1,875 (70.6)	624 (67.2)	*<0.001*	1,491 (80.0)	1,266 (76.2)	1,812 (63.3)	*<0.001*	2,263 (87.9)	2,044 (70.4)	262 (28.9)	*<0.001*
White, *n* (%)	2531 (90.3)	2,496 (94.0)	885 (95.3)	*<0.001*	1,721 (92.4)	1,550 (93.3)	2,641 (92.3)	*0.42*	2,553 (99.1)	2,658 (91.5)	701 (77.5)	*<0.001*
Family history of type 2 diabetes, *n* (%)	291 (10.4)	300 (11.3)	108 (11.6)	*0.35*	175 (9.4)	174 (10.5)	350 (12.2)	*0.007*	228 (8.9)	328 (11.3)	143 (15.8)	*<0.001*
One or more prevalent conditions^b^, *n* (%)	518 (18.5)	551 (20.8)	185 (19.9)	*0.11*	339 (18.2)	301 (18.1)	614 (21.5)	*0.002*	514 (20.0)	559 (19.2)	181 (20.0)	*0.78*
Type 2 diabetes incidence, *n* (rate^c^)	304 (7.6)	317 (8.8)	110 (8.6)	*0.19*	182 (6.9)	188 (8.1)	361 (8.6)	*0.07*	231 (6.0)	358 (9.0)	142 (11.5)	*<0.001*

a *p* for linear trend across socioeconomic categories.

b Prevalent conditions considered are coronary heart disease, stroke, cancer, and hypertension.

c Age, sex, and ethnicity adjusted diabetes incidence rate per 1,000 person-year over a 14.3-y mean follow-up.

CI, 95% CI.

**Figure 1:**
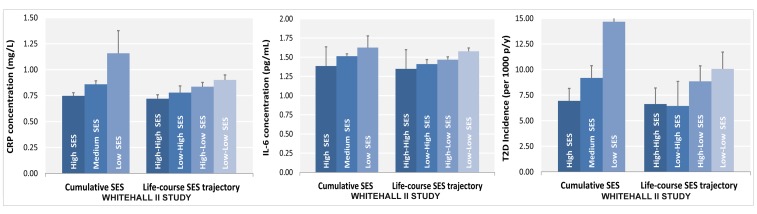
Association of lifecourse socioeconomic status with two inflammatory markers and type 2 diabetes. Lower SES is associated with higher CRP (A) and IL-6 levels (B) and with a greater type 2 diabetes risk (C) after adjustment for sex, age, and ethnicity. All *p* for linear trend between lifecourse SES and inflammatory markers or type 2 diabetes were <0.001. Cumulative SES score includes father's occupational position, participants' education, and participants' occupational position at phase 3. Each SES measure was a 3-level variable with values ranging from 0 (high) to 2 (low). A score was calculated by summing each SES measure (range 0–6). The final cumulative SES score was categorized as high (score = 0–2), middle (score = 3–5), and low (score = 6). Lifecourse SES trajectory refers to father's occupational position and participants' occupational position at phase 3. p-y, person-years; T2D, type 2 diabetes.

Participants in the low versus the high SES group were more likely to be current smokers (odds ratio [OR] = 3.33, 95% CI = 2.52, 4.39 for cumulative SES score), physically inactive (OR = 2.01, 95% CI = 1.58, 2.57 for cumulative SES score), and have an unhealthy diet (OR = 2.80, 95% CI = 2.29, 3.42 for cumulative SES score) for all SES indicators examined (Table 2). They also were more likely to be obese (OR = 1.49, 95% CI = 1.06, 2.09 for cumulative SES score), and have high CRP (OR = 1.61, 95% CI = 1.31, 1.98 for cumulative SES score) and Il-6 (OR = 1.39, 95% CI = 1.13, 1.71 for cumulative SES score) in analyses additionally adjusted for unhealthy behaviors (Table 2). As shown in Figure 1, lower lifecourse SES was associated with an increased incidence of type 2 diabetes (Figure 1C) in a dose-response manner. Similar socioeconomic gradients were also observed for inflammatory markers (CRP, Figure 1A; IL-6, Figure 1B).

**Table 2:** Odds ratios (95% CI) for the association of indicators of socioeconomic status across the lifecourse with type 2 diabetes risk factors at baseline (Whitehall II phase 3), *n* = 6,387.

Type 2 Diabetes Risk Factor	Lowest vs Highest Father's Occupational Position	Lowest vs Highest Education	Lowest vs Highest Adult Occupation	Lowest vs Highest Cumulative SES Score^a^	Low-Low vs High-High SES Trajectory^b^
	OR^c^ (95% CI)	OR^c^ (95% CI)	OR^c^ (95% CI)	OR^c^ (95% CI)	OR^c^ (95% CI)
Current smoking (ref.: never/former smoking)	1.43 (1.17–1.76)	2.22 (1.84–2.69)	3.89 (3.06–4.95)	3.33 (2.52–4.39)	2.78 (2.25–3.44)
Physical inactivity (ref.: physically active)	1.37 (1.14–1.65)	1.31 (1.12–1.54)	2.82 (2.29–3.48)	2.01 (1.58–2.57)	1.75 (1.45–2.10)
Unhealthy diet (ref.: healthy diet)	1.53 (1.32–1.77)	1.80 (1.58–2.04)	2.61 (2.19–3.10)	2.80 (2.29–3.42)	2.25 (1.95–2.60)
Highest carbohydrate intake tertile (ref.: lower carbohydrate intake tertiles)	0.87 (0.75–1.01)	0.79 (0.70–0.89)	0.96 (0.81–1.15)	0.80 (0.65–0.97)	0.87 (0.75–1.00)

	OR^d^ (95% CI)	OR^d^ (95% CI)	OR^d^ (95% CI)	OR^d^ (95% CI)	OR^d^ (95% CI)
BMI≥30 (ref.: BMI<30)	1.30 (1.01–1.67)	1.22 (0.97–1.52)	1.47 (1.10–1.97)	1.49 (1.06–2.09)	1.31 (1.02–1.69)
Highest CRP tertile (ref.: lower CRP tertiles)	1.36 (1.17–1.59)	1.36 (1.19–1.56)	1.36 (1.13–1.63)	1.61 (1.31–1.98)	1.26 (1.08–1.46)
Highest IL-6 tertile (ref.: lower IL-6 tertiles)	1.41 (1.21–1.65)	1.32 (1.15–1.51)	1.33 (1.11–1.60)	1.39 (1.13–1.71)	1.33 (1.14–1.55)

a Cumulative SES score includes father's occupational position, participants' education, and participants' occupational position at phase 3. Each SES measure was a 3-level variable with values ranging from 0 (high) to 2 (low). A score was calculated by summing each SES measure (range 0–6). The final cumulative SES score was categorized as high (score = 0–2), middle (score = 3–5), and low (score = 6).

b Lifecourse SES trajectory refers to father's occupational position and participants' occupational position at phase 3.

c Model adjusted for age, sex, ethnicity, family history of diabetes, and prevalent conditions.

d Model adjusted for age, sex, ethnicity, family history of diabetes, prevalent conditions, smoking, physical activity, diet.

β, Beta coefficient; ref., reference.

Current smoking (HR = 1.56, 95% CI 1.27–1.91), physical inactivity (1.25, 95% CI 1.06–1.48), unhealthy diet (1.42, 95% CI 1.19–1.69), and higher BMI (1.61, 95% CI 1.51–1.72 per 1 SD increase) predicted the development of type 2 diabetes over the follow-up. Higher CRP and IL-6 concentrations were also associated with higher incidence of type 2 diabetes. Multiple adjustments resulted in some attenuation of these estimates but the pattern of association persisted, apart from the association between IL-6 and type 2 diabetes incidence, which was mostly removed (Table 3).

**Table 3:** Hazard ratios (95% CI) for the association of inflammatory markers and other risk factors with type 2 diabetes incidence (*n* = 6,387; 731 incident diabetes cases).

Type 2 Diabetes Risk Factors	HR^a^ (95% CI)	HR^b^ (95% CI)
**Smoking**		
Never/former smoker	1.00 (ref.)	1.00 (ref.)
Current smoker	1.56 (1.27–1.91)	1.34 (1.09–1.66)
**Physical activity**		
Active	1.00 (ref.)	1.00 (ref.)
Moderately active	1.19 (0.98–1.45)	1.00 (0.81–1.22)
Inactive	1.25 (1.06–1.48)	1.20 (1.01–1.42)
**Diet (AHEI)**		
Healthy	1.00 (ref.)	1.00 (ref.)
Moderately healthy	1.00 (0.83–1.20)	0.90 (0.75–1.09)
Unhealthy	1.42 (1.19–1.69)	1.24 (1.03–1.49)
**Carbohydrate intake**		
Low	1.00 (ref.)	1.00 (ref.)
Middle	0.88 (0.74–1.06)	1.00 (0.83–1.19)
High	0.90 (0.75–1.09)	1.08 (0.89–1.30)
**BMI**		
1 SD increase	1.61 (1.51–1.72)	1.49 (1.39–1.60)
**CRP**		
1 SD increase	1.59 (1.46–1.73)	1.32 (1.19–1.45)
**IL-6**		
1 SD increase	1.25 (1.16–1.34)	1.02 (0.93–1.11)

a Model adjusted for age, sex, ethnicity, family history of diabetes, and prevalent conditions.

b Model adjusted for age, sex, ethnicity, family history of diabetes, prevalent conditions, and mutually adjusted for all risk factors.

ref., reference.

Participants with a middle and low occupational position of the father or with a middle or low personal education had an increased risk of developing type 2 diabetes over the follow-up compared with those whose father had a high occupational position or whose education was high (HR = 1.17, 95% CI = 1.00–1.37 for middle versus high father's occupational position and HR = 1.28, 95% CI = 1.07–1.54 for low versus high education). Adult occupational position was strongly associated with type 2 diabetes incidence (Table 4). Smoking, physical activity, diet, and BMI explained 69% (95% CI 26–801) of the association between medium father's occupational position and type 2 diabetes, 60% (95% CI 28–240) of the association between low education and type 2 diabetes, and 39% (95% CI 22–75) of the association between low adult occupational position and type 2 diabetes. Inflammatory markers explained 40% (95% CI 14–463) of the association between father's occupational position and type 2 diabetes; 36% (95% CI 17–133) of that between education and type 2 diabetes; and 26% (95% CI 16–50) of that between occupational position and type 2 diabetes (Table 4).

**Table 4:** Hazard ratios (95% CI) for the association of indicators of socioeconomic status in early and adult life with type 2 diabetes incidence (*n* = 6,387; 731 incident diabetes cases).

Models	Father's Occupational Position					Education					Adult Occupational Position				
	High	Medium		Low		High	Medium		Low		High	Medium		Low	
	HR (ref)	HR (95% CI)	%Δ (95% CI)	HR (95% CI)	%Δ (95% CI)	HR (ref)	HR (95% CI)	%Δ (95% CI)	HR (95% CI)	%Δ (95% CI)	HR (ref)	HR (95% CI)	%Δ (95% CI)	HR (95% CI)	%Δ (95% CI)
**Model 1** a	1.00	1.17 *(1.00–1.37)*	ref	1.15 *(0.92–1.43)*		1.00	1.18 *(0.96–1.45)*		1.28 *(1.07–1.54)*	ref	1.00	1.41 *(1.18–1.67)*		1.87 *(1.46–2.40)*	ref
**Model 2:** Model 1+smoking, physical activity, diet, BMI^b^		1.05 *(0.89–1.23)*	−69 *(−801 to −26)*	1.05 *(0.84–1.31)*	N/A		1.08 *(0.88–1.33)*	N/A	1.11 *(0.92–1.33)*	−60 *(−240 to −28)*		1.30 *(1.09–1.55)*	−23 *(−53 to −9)*	1.46 *(1.13–1.90)*	−39 *(−75 to −22)*
**Model 3:** Model 1+CRP^b^		1.11 *(0.94–1.30)*	−34 *(−377 to −11)*	1.06 *(0.85–1.33)*	N/A		1.14 *(0.93–1.40)*	N/A	1.19 *(0.99–1.43)*	−30 *(−115 to −13)*		1.31 *(1.10–1.56)*	−20 *(−44 to −11)*	1.62 *(1.26–2.08)*	−23 *(−43 to −14)*
**Model 4:** Model 1+IL-6^b^		1.13 *(0.96–1.33)*	−20 *(−209 to −6)*	1.08 *(0.87–1.35)*	N/A		1.17 *(0.95–1.43)*	N/A	1.21 *(1.01–1.46)*	−22 *(−85 to −10)*		1.34 *(1.13–1.59)*	−14 *(−36 to −8)*	1.74 *(1.35–2.23)*	−12 *(−23 to −7)*
**Model 5:** Model 1+CRP +IL-6^b^		1.10 *(0.94–1.29)*	−40 *(−463 to −14)*	1.05 *(0.84–1.31)*	N/A		1.14 *(0.93–1.40)*	N/A	1.17 *(0.97–1.41)*	−36 *(−133 to −17)*		1.29 *(1.09–1.54)*	−24 *(−58 to −14)*	1.59 *(1.23–2.04)*	−26 *(−50 to −16)*
**Model 6:** Model 1+all risk factors^b^		1.03 *(0.88–1.21)*	−77 *(−901 to −29)*	1.02 *(0.82–1.28)*	N/A		1.09 *(0.88–1.33)*	N/A	1.09 *(0.90–1.31)*	−65 *(−250 to −32)*		1.27 *(1.06–1.51)*	−31 *(−69 to −15)*	1.42 *(1.09–1.84)*	−44 *(−81 to −27)*
Additional contribution of CRP+IL-6 to Model 2			**−32** c							**−14** c			**−10** c		**−8** c

a Adjusted for age, sex, ethnicity, family history of diabetes, and prevalent conditions.

b All risk factors are updated at phases 3, 5, and 7 and additionally adjusted for the risk factor at the previous phase.

c Additional contribution of CRP and IL-6 to the model adjusted for age, sex, ethnicity, family history of diabetes, prevalent conditions, smoking, physical activity, BMI, and diet.

Δ, attenuation; N/A, not applicable; ref., reference.

In Table 5, the association between the cumulative SES score and type 2 diabetes incidence is presented. In analyses adjusted for age, sex, ethnicity, family history of diabetes, and prevalent conditions, participants with the lowest cumulative SES score (i.e., the most deprived) had almost double the risk (1.96; 95% CI = 1.48–2.58) of developing diabetes during follow-up than participants with the highest cumulative SES score. BMI accounted for around 20% (95% CI 11–41) of this association and all behavioral factors together for 34% (95% CI 20–68). Inflammatory markers explained 25% (95% CI 16–48) of the association between low cumulative SES score and type 2 diabetes incidence, of which about 40% (i.e., 10%) was independent from behavioral risk factors. After adjustment for all behavioral and inflammatory risk factors, the HR for the lowest versus the highest cumulative SES score was reduced to 1.49 (95% CI = 1.12–1.99), a 40% (95% CI 24–78) attenuation.

**Table 5:** Hazard ratios (95% CI) for the association of cumulative socioeconomic score with type 2 diabetes incidence (*n* = 6,387; 731 incident diabetes cases).

Models	Cumulative SES Score^a^	
	HR (95% CI)	%Δ (95% CI)
**Model 1:** Adjusted for age, sex, ethnicity family history, and prevalent conditions	1.96 (1.48–2.58)	ref.
**Model 2:** Model 1+smoking^b^	1.88 (1.42–2.49)	−6 (−15 to −1)
**Model 3:** Model 1+physical activity^b^	1.91 (1.44–2.52)	−4 (−12 to −1)
**Model 4:** Model 1+diet^b^	1.84 (1.39–2.44)	−9 (−20 to −4)
**Model 5:** Model 1+BMI^b^	1.71 (1.29–2.27)	−20 (−41 to −11)
**Model 6:** Model 1+smoking, physical activity, diet, and BMI^b^	1.56 (1.17–2.07)	−34 (−68 to −20)
**Model 7:** Model 1+CRP^b^	1.69 (1.28–2.24)	−22 (−41 to −13)
**Model 8:** Model 1+IL-6^b^	1.79 (1.35–2.37)	−13 (−27 to −8)
**Model 9:** Model 1+CRP +IL-6^b^	1.65 (1.25–2.18)	−25 (−48 to −16)
**Model 10:** Model 1+all risk factors^b^	1.49 (1.12–1.99)	−40 (−78 to −24)
Additional contribution of CRP+IL-6 to Model 5^b^		**−10** c

a The cumulative SES score is entered as a continuous 3-level variable into the models. HR is for the lowest versus highest score.

b All risk factors are updated at phases 3, 5, and7 and additionally adjusted for the risk factor at the previous phase.

c Additional contribution of CRP and IL-6 to the model adjusted for age, sex, ethnicity, family history of diabetes, prevalent conditions, smoking, physical activity, BMI, and diet.

ref., reference; Δ, attenuation.

**Figure 2:**
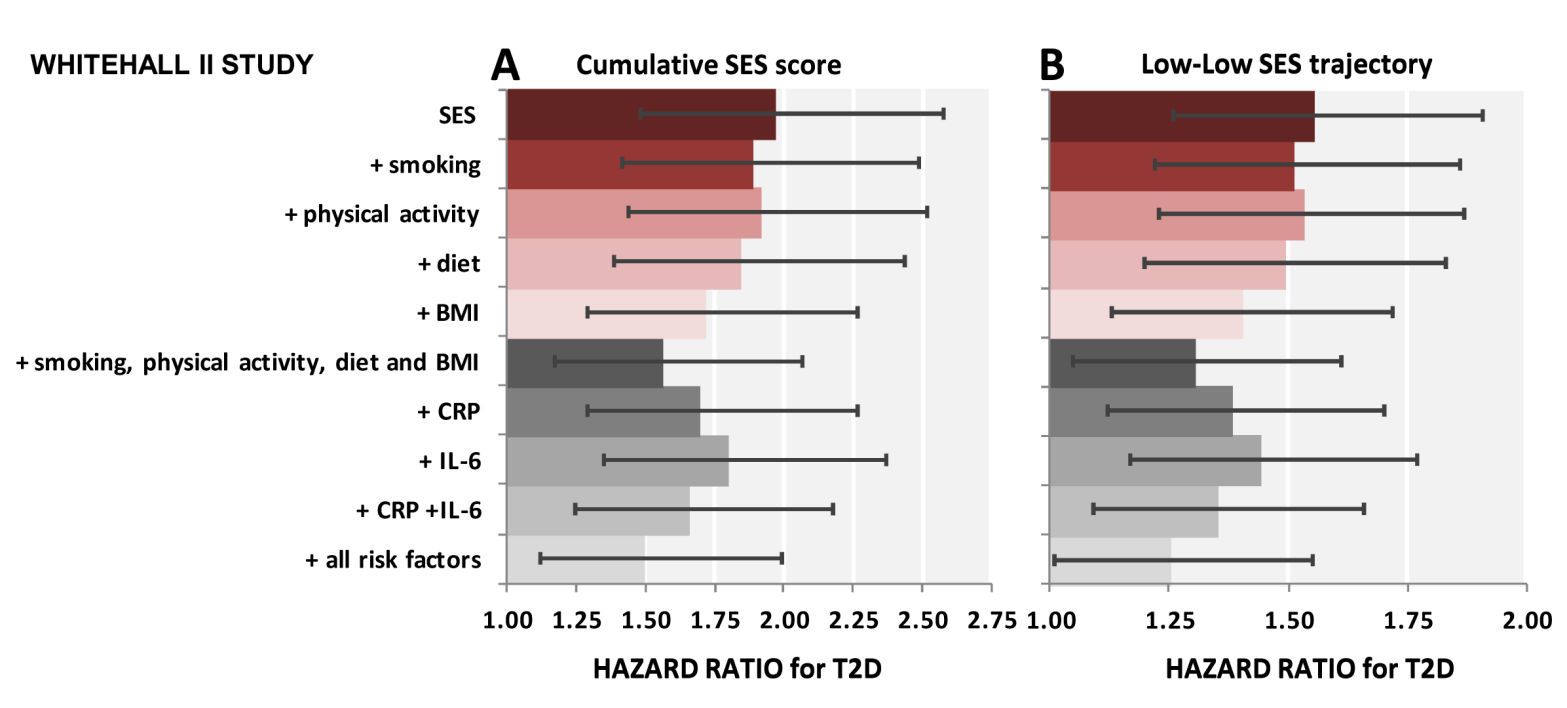
Contribution of smoking, physical activity, diet, BMI, CRP, and IL-6 to the association between lifecourse socioeconomic status and type 2 diabetes incidence. The first bar shows explanatory factors for the associations of low cumulative SES score (ref. high cumulative SES score) (A) and adverse SES-trajectory (ref. high-high SES trajectory) (B) with type 2 diabetes (T2D). Inflammatory markers, in combination, explain 26% (95% CI 16%–46%) of the first association (A) and 34% (95% CI 20%–62%) of the latter association (B). All associations are adjusted for age, sex, ethnicity, family history of T2D, and prevalent conditions. Cumulative SES score includes father's occupational position, participants' education, and participants' occupational position at phase 3. Each SES measure was a 3-level variable with values ranging from 0 (high) to 2 (low). A score was calculated by summing each SES measure (range 0–6). The final cumulative SES score was categorized as high (score = 0–2), middle (score = 3–5), and low (score = 6). Lifecourse SES trajectory refers to father's occupational position and participants' occupational position at phase 3.

Table 6 shows results for the association between lifetime SES trajectories and type 2 diabetes incidence. Participants with low SES in childhood but high SES in adulthood were not at a higher risk of type 2 diabetes than participants with a stable high SES trajectory. Participants who were socially downwardly mobile (high SES in childhood and low SES in adulthood) or had low SES in both childhood and adulthood had, respectively, a 1.4- and a 1.6-fold increased risk of developing type 2 diabetes over the follow-up compared to those who had high SES in both childhood and adulthood (95% CI 1.11–1.69 for high-low trajectory and 95% CI1.26–1.91 for low-low trajectory). Inflammatory markers explained about 30% of this increased risk (95% CI 10–60), of which about 40% (i.e., 12% for high-low and 14% for low-low trajectory) was independent of behavioral risk factors. The contribution of risk factors to the association of cumulative SES score and low-low SES trajectory from childhood to adulthood with type 2 diabetes incidence is also illustrated in Figure 2.

**Table 6:** Hazard ratios (95% CI) for the association of lifecourse socioeconomic trajectories with type 2 diabetes incidence (*n* = 6,387; 731 incident diabetes cases).

Models	SES Trajectory					
	Low-High Vs. High-High		High-Low Vs. High-High		Low-Low Vs. High-High	
	HR (95% CI)	%Δ (95% CI)	HR (95% CI)	%Δ (95% CI)	HR (95% CI)	%Δ (95% CI)
**Model 1:** Adjusted for age, sex, ethnicity, family history, prevalent conditions	0.99 (0.75–1.31)		1.37 (1.11–1.69)	ref.	1.55 (1.26–1.91)	ref.
**Model 2:** Model 1+smoking^a^	0.98 (0.74–1.30)	N/A	1.34 (1.09–1.65)	−7 (−18 to −1)	1.51 (1.22–1.86)	−7 (−16 to −1)
**Model 3:** Model 1+physical activity^a^	0.99 (0.75–1.31)	N/A	1.35 (1.09–1.66)	−6 (−22 to −2)	1.51 (1.23–1.87)	−6 (−15 to −2)
**Model 4:** Model 1+diet^a^	0.97 (0.73–1.28)	N/A	1.32 (1.07–1.63)	−12 (−28 to −1)	1.49 (1.20–1.83)	−10 (−19 to −2)
**Model 5:** Model 1+BMI^a^	0.98 (0.73–1.28)	N/A	1.37 (1.11–1.69)	0 (−10 to 18)	1.40 (1.13–1.72)	−24 (−46 to −11)
**Model 6:** Model 1+smoking, physical activity, diet and BMI^a^	0.94 (0.71–1.25)	N/A	1.29 (1.04–1.59)	−19 (−46 to 0)	1.30 (1.05–1.61)	−40 (−72 to −20)
**Model 7:** Model 1+CRP^a^	0.94 (0.72–1.25)	N/A	1.27 (1.03–1.57)	−23 (−51 to −9)	1.38 (1.12–1.70)	−27 (−49 to −15)
**Model 8:** Model 1+IL-6^a^	0.97 (0.73–1.28)	N/A	1.32 (1.07–1.62)	−13 (−31 to −4)	1.44 (1.17–1.77)	−18 (−32 to −9)
**Model 9:** Model 1+CRP +IL-6^a^	0.94 (0.71–1.24)	N/A	1.26 (1.02–1.55)	−27 (−60 to −10)	1.35 (1.09–1.66)	−32 (−58 to −18)
**Model 10:** Model 1+all risk factors^a^	0.92 (0.70–1.22)	N/A	1.25 (1.01–1.54)	−29 (−65 to −7)	1.25 (1.01–1.55)	−48 (−87 to −25)
Additional contribution of CRP+IL-6 to Model 5				**−12** b		**−14** b

a All risk factors are updated at phases 3, 5, and 7 and additionally adjusted for the risk factor at the previous phase.

b Additional contribution of CRP and IL-6 to the model adjusted for age, sex, ethnicity, family history of diabetes, prevalent conditions, smoking, physical activity, BMI, and diet.

N/A, not applicable; ref., reference; Δ, attenuation.

The associations of indicators of SES in early life and downward socioeconomic trajectory with type 2 diabetes incidence were almost completely accounted for by adjustment for adult occupational position or cumulative SES score. Adult occupational position and cumulative SES score were independently related to type 2 diabetes incidence (Table S5).

### Sensitivity Analyses

All analyses were repeated in subgroups including participants with complete data (Table S3); furthermore, adjustments were extended to include factors such as pack years of cigarettes smoked, alcohol consumption, drug intake, height, and BMI in early adulthood (25 y of age). These sensitivity analyses yielded similar results to those reported in the main analysis (Table S6). Analyses were also repeated using age as the time scale instead of time-to-event in Cox regressions; results did not vary (Table S7). We tested whether there was a modification effect by gender or ethnicity in the association between lifecourse SES and type 2 diabetes, and found no evidence for such an effect (*p* for interaction >0.05).

We additionally assessed whether missing values at baseline could have biased our results using multiple multivariate imputation to replace missing values for risk factors at the study baseline (STATA ice/micombine procedures). Analyses on the imputed dataset (*n* = 8,526, 909 incident type 2 diabetes cases) were similar to those reported in the main analysis (Table S8).

Finally, in our study incident type 2 diabetes was being predicted by inflammatory markers assessed premorbidly (5 to 10 y prior to the onset of type 2 diabetes). However, a residual confounding of insulin resistance on inflammatory markers could not be completely excluded as insulin resistance can be present several years before diabetes onset [56]. We repeated all analyses on participants with more than 10 y of follow-up (*n* = 5,282, 440 incident diabetes cases), allowing for a gap of more than 10 y between inflammatory markers assessment and type 2 diabetes onset. The contribution of inflammation to the lifecourse SES-type 2 diabetes association was similar to that presented in the main analysis (for the cumulative SES score 24% versus 27%in the main analysis and for downward SES trajectory 25% versus 32% in main analysis).

## Discussion

Adverse socioeconomic circumstances in early and later life have been related to an increased risk of metabolic disorders in adulthood, but the mechanisms underlying this link remain poorly understood. Building on recent animal models, we hypothesized that chronic inflammation might partly explain the link between lifecourse SES and type 2 diabetes. Our findings from a large population study confirmed this. First, we found that cumulative exposure to low SES over the lifecourse and a downward trajectory from high SES in childhood to low SES in adulthood were robustly associated with an increased risk of developing type 2 diabetes over the study period. Second, inflammatory processes, measured repeatedly through CRP and interleukin-6, contributed to explain as much as one third of this association.

This evidence should be interpreted cautiously. As noted earlier, early life socioeconomic circumstances can have an impact on health in adulthood because of latent effects of early-life circumstances on adult health, independent of socioeconomic conditions in adult life; through cumulative effects whereby the duration of exposure to adverse socioeconomic circumstances from across the lifecourse affects health in a dose-response manner; or because of pathway effects by which early life socioeconomic circumstances affect the individuals' trajectories to SES in later life, that in turn have an impact on health [44]. In relation to type 2 diabetes incidence, previous studies reported an association between duration of exposure to socioeconomic adversity, as well as a downward socioeconomic trajectory, and increased risk of type 2 diabetes [45],[57],[58], consistently with our findings. In contrast, results for an independent effect of childhood SES on adult incidence of type 2 diabetes have been inconsistent [8],[45],[57]–[59]. Our study does not support the hypothesis that early-life SES would affect type 2 diabetes in adulthood independently of SES in adult life.

In our study, participants with low lifecourse SES had increased CRP and IL-6 concentrations compared with participants with high lifecourse SES. These data are consistent with previous studies reporting associations between SES and inflammatory markers [30],[60],[61], including one report from the Whitehall II cohort [62]. Our study is consistent with the hypothesis that socioeconomic differences in inflammatory activity might explain a considerable proportion of socioeconomic differences in inflammation-related diseases, such as type 2 diabetes [32]. Importantly, we found that only socioeconomic trajectories including low adult SES were associated with increased type 2 diabetes risk. This suggests that the adverse effects of low SES in early life might be possible to reverse by favorable socioeconomic circumstances in adulthood, a finding that is consistent with a recent animal study [25] demonstrating that SES-related regulatory changes may also still occur in adulthood.

**Figure 3:**
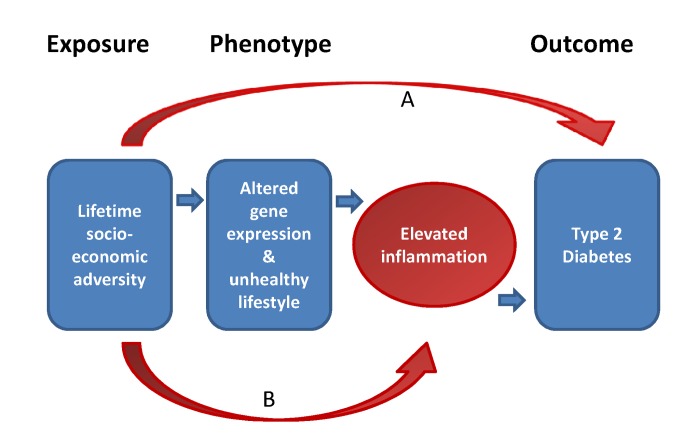
Simplified conceptual framework for the potential role of inflammatory processes in explaining social inequalities in type 2 diabetes. Socioeconomic adversity over the lifetime is hypothesized to be associated with type 2 diabetes risk. Part of this association might be mediated by the elevated inflammatory states resulting from altered gene expression and/or unhealthy lifestyles, both related to socioeconomic adversity. Other factors (e.g., low birth weight) may also mediate part of the association between SES and type 2 diabetes (arrow A). Furthermore, SES is hypothesized to contribute to elevated inflammation because of comorbid conditions (arrow B).

Diverse biological mechanisms are likely to contribute to the mediating role of inflammation in the lifecourse SES-type 2 diabetes association (Figure 3). First, SES could affect inflammation through stress-mediated factors involving the hypothalamic-pituitary-adrenal axis and the autonomic nervous system [26]–[28],[30],[63]–[65]. Recent evidence reporting SES-related epigenetic changes in genomic regions regulating response to stress supports this possibility [20]–[22],[25]. SES differences in gene regulation of response to stress can be reflective of environmental/dietary exposures occurring over the lifecourse or be a direct consequence of developmental programming in early life.

Second, lifestyle factors can underlie the associations between inflammation, lifecourse SES, and type 2 diabetes because inflammatory processes are related to several type 2 diabetes risk factors. These include smoking, obesity, unhealthy diet, and physical inactivity, all of which show strong socioeconomic gradients [66]–[70]. We found that more than half of the contribution of inflammation to SES differences in type 2 diabetes was attributable to the pro-inflammatory effect of smoking, physical inactivity, unhealthy diet, and obesity. In addition, chronic stress associated with socioeconomic adversity may influence at the same time lifestyle factors and inflammatory activity [71].

Several other determinants of inflammation, such as subclinical diseases (atherosclerosis and coronary heart disease) or medication use are also socially patterned [72]–[74] and could account for part of the associations between SES, inflammation, and type 2 diabetes. However, our results were adjusted for prevalent conditions, such as coronary heart disease, stroke, cancer, and hypertension, and additional adjustment for drug intake did not impact our findings. Finally, further work involving assessment of SES-related changes in gene regulation, of additional inflammatory markers, and of more precise measures of adiposity will be necessary in order to gain better understanding of the pathophysiological and biochemical processes linking SES, inflammation, and type 2 diabetes.

Some limitations to this study are noteworthy. First, the participants were from an occupational cohort, which is not representative of the general population with regards to the socioeconomic spectrum included and the prevalence of risk factors observed. In particular, people who experienced extreme social adversity in early life and eventually ended up with temporary jobs or unemployed are not represented in this study. Second, our measure of socioeconomic circumstances in early-life, father's occupational position, was collected retrospectively. Misclassification of father's occupation may lead to under- or over-estimation of its true effect on adult health [75],[76]. In the same way, our composite measures of SES rely on the assumption that SES indicators are measured with the same precision across the lifespan. The potential misclassification issues related to father's occupation might in part explain the weaker impact of early life SES on type 2 diabetes incidence.

Third, studies have suggested that the association between SES indicators in adulthood and disease risk might in part be explained by health-related selection into lower social classes. A previous report using data from this cohort study, for example, suggested that health-related selection indeed operates at younger ages, although it may contribute less to socioeconomic differences in cardio-metabolic health in midlife [77]. In the present study, reverse causation between SES and type 2 diabetes is an unlikely explanation for the findings as none of the participants were diabetic at the time of the measurement of SES. However, the effect of other morbidity on both educational attainment and occupational position cannot be ruled out and this might in part explain the association between declining SES trajectory and increased risk of developing type 2 diabetes. Finally, health behaviours were self-reported and it has been shown that questionnaire-based measures are not entirely valid [78],[79]. However, data on smoking, diet, and physical activity were collected using standard questions that have been validated against objectively measured outcomes in previous studies [80]–[82].

This study has also important strengths. To our knowledge, it is one of the first studies to examine the contribution of inflammation to the association between lifecourse SES and type 2 diabetes incidence. We assessed current and long-term exposure to raised levels of inflammatory markers over a long follow-up; we are not aware of other studies with such repeat measurements combined with type 2 diabetes follow-up through OGTTs. This is important as it allowed us to account for long-term exposure to increased inflammatory activity in relation to objective measurement of type 2 diabetes incidence.

Our findings have several implications. First, these data provide new evidence that inflammation might contribute to explain a substantial part of the association between duration of exposure to socioeconomic adversity and increased incidence of type 2 diabetes. Further studies are needed, particularly on population-based samples, to confirm our findings. Second, our findings extend previous results from animal models on social rank and inflammation, and are consistent with the idea that SES might affect regulation of inflammation-related genes. Future (epi)genetic research is needed to test this possibility in humans. Third, this study demonstrates the importance of using repeated measurements of exposures over time to assess the contribution of long-term inflammation to social inequalities in type 2 diabetes. Fourth, assuming that our findings reflect a causal association, our results suggest that tackling socioeconomic differences in inflammation, especially among the most disadvantaged groups, might reduce social inequalities in type 2 diabetes. However, intervention studies will be necessary to determine the extent to which social inequalities attributable to chronic inflammation are reversible. Interventions known to reduce inflammation and diabetes risk include, for example, weight management, physical activity, and smoking cessation programs. Furthermore, anti-inflammatory drugs are currently being studied for primary prevention of site-specific cancers [83]–[85], although little evidence is available in relation to type 2 diabetes.

## Supporting Information

Figure S1 **Simplified representation of the study design.** T2D, type 2 diabetes.
(TIF)

Table S1 **Spearman's correlation matrix between indicators of socioeconomic status.**
(DOCX)

Table S2 **Missing values.**
(DOCX)

Table S3 **Association of cumulative socioeconomic score with type 2 diabetes incidence (** ***n*** **= 5,923; 488 incident diabetes cases).** Complete case analysis.
(DOCX)

Table S4 **Comparison of included and excluded participants on selected indicators.**
(DOCX)

Table S5 **Association of socioeconomic indicators with type 2 diabetes incidence (** ***n*** **= 6,347; 731 incident diabetes cases).**
(DOCX)

Table S6 **Association of cumulative socioeconomic score with type 2 diabetes incidence (** ***n*** **= 6,198; 641 incident diabetes cases).** Additional adjustment for pack years of cigarettes smoked, drug intake, alcohol consumption, height, and BMI at 25 y.
(DOCX)

Table S7 **Association of cumulative socioeconomic score with type 2 diabetes incidence (** ***n*** **= 6,387; 731 incident diabetes cases).** Age as the time scale.
(DOCX)

Table S8 **Association of cumulative socioeconomic score with type 2 diabetes incidence (** ***n*** **= 8,526; 909 incident diabetes cases).** Multiple imputation (STATA ICE/micombine procedures).
(DOCX)

## Abbreviations

AHEI: Alternative Healthy Eating Index
BMI: body mass index
CRP: C-reactive protein
HR: hazard ratio
IL-6: interleukin 6
OGTT: oral glucose tolerance test
OR: odds ratio
SD: standard deviation
SES: socioeconomic status
