# Supplementary figures and images for: Association of Lifecourse Socioeconomic Status with Chronic Inflammation and Type 2 Diabetes Risk: The Whitehall II Prospective Cohort Study
Source: PLoS Med. 2013 Jul 2;10(7):e1001479. doi: 10.1371/journal.pmed.1001479 (PMC3699448; doi:10.1371/journal.pmed.1001479)

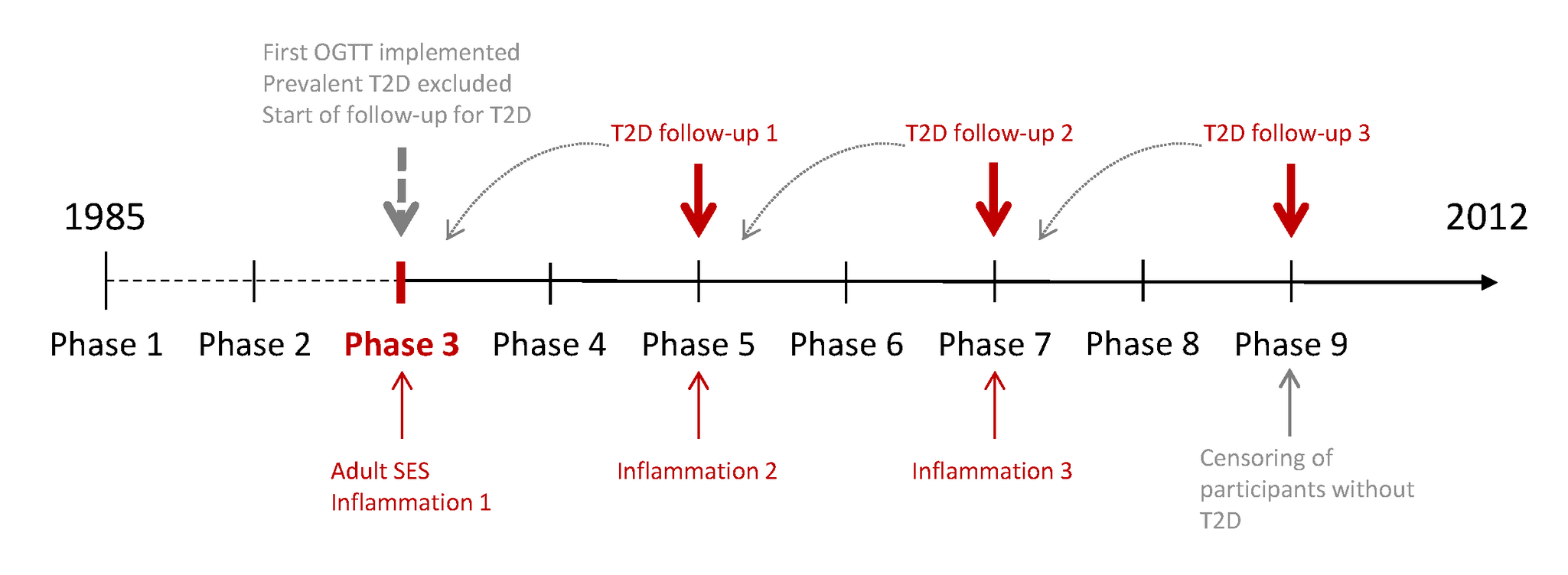

Supplement: Figure S1 — Simplified representation of the study design. T2D, type 2 diabetes. (TIF) [file pmed.1001479.s001.tif]
