# Supplementary material for: Association of Lifecourse Socioeconomic Status with Chronic Inflammation and Type 2 Diabetes Risk: The Whitehall II Prospective Cohort Study
Source: PLoS Med. 2013 Jul 2;10(7):e1001479. doi: 10.1371/journal.pmed.1001479 (PMC3699448; doi:10.1371/journal.pmed.1001479)
Supplement: Table S1 — Spearman's correlation matrix between indicators of socioeconomic status. (DOCX) [file pmed.1001479.s002.docx]

**Table S1. Spearman’s correlation matrix between indicators of socioeconomic status**

|  | Father’s occupation | Education | Adult occupation | Cumulative SES score | SES trajectory |
| --- | --- | --- | --- | --- | --- |
| Father’s occupation | 1 |  |  |  |  |
| Education | 0.2676* | 1 |  |  |  |
| Adult occupation | 0.1764* | 0.4463* | 1 |  |  |
| Cumulative SES score | 0.5252* | 0.7617* | 0.6251* | 1 |  |
| SES trajectory | 0.4767* | 0.4656* | 0.8429* | 0.6831* | 1 |

SES: Socioeconomic status

* indicates significance at p=0.05
