# Supplementary material for: Association of Lifecourse Socioeconomic Status with Chronic Inflammation and Type 2 Diabetes Risk: The Whitehall II Prospective Cohort Study
Source: PLoS Med. 2013 Jul 2;10(7):e1001479. doi: 10.1371/journal.pmed.1001479 (PMC3699448; doi:10.1371/journal.pmed.1001479)
Supplement: Table S2 — Missing values. (DOCX) [file pmed.1001479.s003.docx]

**Table S2. Missing values**

|  | **Phase 3 (N=8815)** | | **Phase 5 (N=7829)** | | **Phase 7 (N=6986)** | |
| --- | --- | --- | --- | --- | --- | --- |
|  | *Missing* | *Missing after imputation* | *Missing* | *Missing after imputation* | *Missing* | *Missing after imputation* |
| Family history | 177 | Not imputed |  |  |  |  |
| Education | 696 | Not imputed |  |  |  |  |
| Father’s occupation | 754 | Not imputed |  |  |  |  |
| Diabetes status | 529 | Not imputed | 835 | Not imputed | 599 | Not imputed |
| C-reactive protein ^b^ | 1208 | **721** | 1577 | **354** | 804 | **143** |
| Interleukin-6 ^b^ | 1261 | **723** | 1550 | **352** | 1259 | **146** |
| Smoking status^c^ | 502 | **0** | 599 | **0** | 0 | **0** |
| Physical activity^c^ | 496 | **15** | 750 | **6** | 186 | **0** |
| Diet^c^ | 486 | **5** | 333 | **3** | 132 | **2** |
| Body Mass Index^c^ | 741 | **5** | 2183 | **2** | 518 | **2** |

NA: Not available

Note: There were no missing values for age, sex, ethnicity, adult occupation, prevalent CHD, stroke and cancer.

^a^ Replaced with information collected at previous phase.

^b^ Imputed using multivariate imputation based on sex, age, ethnicity, BMI, health behaviors and, for phases 5 and 7, also on inflammatory markers at the preceding phase.

^c^ Replaced with information collected at previous or successive phases.
