# Supplementary material for: Association of Lifecourse Socioeconomic Status with Chronic Inflammation and Type 2 Diabetes Risk: The Whitehall II Prospective Cohort Study
Source: PLoS Med. 2013 Jul 2;10(7):e1001479. doi: 10.1371/journal.pmed.1001479 (PMC3699448; doi:10.1371/journal.pmed.1001479)
Supplement: Table S3 — Association of cumulative socioeconomic score with type 2 diabetes incidence ( n = 5,923; 488 incident diabetes cases). Complete case analysis. (DOCX) [file pmed.1001479.s004.docx]

**Table S3. Association of cumulative socioeconomic score with type 2 diabetes incidence (N=5923; 488 incident diabetes cases). COMPLETE CASE ANALYSIS**

| **Cumulative SES score^a^** | **HR (95%CI)** | **%Δ** |
| --- | --- | --- |
| **Model 1:** Adjusted for age, sex, ethnicity family history and prevalent conditions | 1.94 (1.38-2.73) | Ref. |
| **Model 8:** Model 1 + smoking, physical activity, diet and BMI^b^ | 1.59 (1.12-2.25) | -30 |
| **Model 11:** Model 1 + CRP +IL-6^b^ | 1.64 (1.17-2.31) | -35 |
| **Model 12:** Model 1 + all risk factors^b^ | 1.52 (1.08-2.15) | -37 |
| Additional contribution of CRP+IL-6 to Model 5^b^ |  | **-9^c^** |

BMI: Body Mass Index; CI: Confidence Interval; CRP: C - reactive protein; HR: Hazard Ratio; IL-6: Interleukin-6; Ref: Reference; SES: Socioeconomic Status; Δ: Attenuation

^a^ The cumulative SES score is entered as a continuous 3-level variable into the models. Hazard ratio is for the lowest vs. highest score.

^b^ All risk factors are updated at Phases 3, 5 &7 and additionally adjusted for the risk factor at the previous phase.

^c^Additional contribution of CRP and IL-6 to the model adjusted for age, sex, ethnicity, family history of diabetes, prevalent conditions, smoking, physical activity, diet, and BMI.
