# Supplementary material for: Association of Lifecourse Socioeconomic Status with Chronic Inflammation and Type 2 Diabetes Risk: The Whitehall II Prospective Cohort Study
Source: PLoS Med. 2013 Jul 2;10(7):e1001479. doi: 10.1371/journal.pmed.1001479 (PMC3699448; doi:10.1371/journal.pmed.1001479)
Supplement: Table S4 — Comparison of included and excluded participants on selected indicators. (DOCX) [file pmed.1001479.s005.docx]

**Table S4. Comparison of included and excluded participants on selected indicators.**

|  | Included | Excluded | *p* ^a^ |
| --- | --- | --- | --- |
| N (%) | 6387 (73.9) | 2259 (26.1) |  |
| Men, N (%) | 4570 (71.5) | 1390 (63.5) | *<0.001* |
| Age, Mean (SD) | 49.3 (6.0) | 51.0 (6.2) | *<0.001* |
| Low adult occupation, N (%) | 910 (14.2) | 602 (26.7) | *<0.001* |
| Type 2 diabetes incidence, N (Rate^b^) | 733 (8.3) | 167 (7.8) | =0.147 |

SD: Standard deviation; CI: 95% Confidence Interval

^a^ p for chi-squared test or test for trend (as appropriate)

^b^ Age, sex and ethnicity adjusted diabetes incidence rate per 1000 person-year over a 14.3 years mean follow-up
