# Supplementary material for: Association of Lifecourse Socioeconomic Status with Chronic Inflammation and Type 2 Diabetes Risk: The Whitehall II Prospective Cohort Study
Source: PLoS Med. 2013 Jul 2;10(7):e1001479. doi: 10.1371/journal.pmed.1001479 (PMC3699448; doi:10.1371/journal.pmed.1001479)
Supplement: Table S5 — Association of socioeconomic indicators with type 2 diabetes incidence ( n = 6,347; 731 incident diabetes cases). (DOCX) [file pmed.1001479.s006.docx]

**Table S5. Association of socioeconomic indicators with type 2 diabetes incidence (N=6347; 731 incident diabetes cases)**

| **SES indicators** | **Model 1^a^** | **Model 2: Model 1 + adult SES** | **Model 3: Model 1 + cumulative SES score^b^** | **Model 2 + lifestyle factors^c^** | **Model 3 + lifestyle factors^c^** |
| --- | --- | --- | --- | --- | --- |
|  | **HR (95%CI)** | **HR (95%CI)** | **HR (95%CI)** | **HR (95%CI)** | **HR (95%CI)** |
| **Father’s occupation** |  |  |  |  |  |
| High | 1.00 | 1.00 | 1.00 | 1.00 | 1.00 |
| Middle | 1.17 (1.00-1.37) | 1.09 (0.93-1.29) | 1.00 (0.79-1.24) | 1.00 (0.86-1.19) | 0.85 (0.80-1.13) |
| Low | 1.15 (0.92-1.43) | 1.06 (0.85-1.33) | 0.86 (0.67-1.13) | 0.99 (0.79-1.24) | 0.85 (0.66-1.10) |
| **Education** |  |  |  |  |  |
| High | 1.00 | 1.00 | 1.00 | 1.00 | 1.00 |
| Middle | 1.18 (0.96-1.45) | 1.07 (0.87-1.32) | 1.00 (0.80-1.24) | 1.00 (0.81-1.24) | 0.94 (0.75-1.18) |
| Low | 1.28 (1.07-1.54) | 1.07 (0.87-1.31) | 0.86 (0.66-1.12) | 0.97 (0.79-1.19) | 0.82 (0.63-1.07) |
| **Adult occupation** |  |  |  |  |  |
| High | 1.00 |  | 1.00 |  | 1.00 |
| Middle | 1.41 (1.18-1.67) |  | 1.27 (1.04-1.54) |  | 1.19 (0.98-1.46) |
| Low | 1.87 (1.46-2.40) |  | 1.56 (1.15-2.11) |  | 1.29 (0.94-1.76) |
| **Cumulative SES score^b^** | 1.96 (1.48-2.58) | 1.44 (1.02-2.06) |  | 1.29 (0.91-1.80) |  |
| **SES trajectories** |  |  |  |  |  |
| High-High | 1.00 | 1.00 | 1.00 |  |  |
| Low-High | 0.99 (0.75-1.31) | 0.99 (0.75-1.31) | 0.89 (0.66-1.19) | 0.94 (0.71-1.25) | 0.87 (0.65-1.17) |
| High-Low | 1.37 (1.11-1.69) | 0.90 (0.75-1.09) | 1.19 (0.94-1.51) | 1.00 (0.83-1.20) | 1.15 (0.90-1.45) |
| Low-Low | 1.55 (1.26-1.91) | 1.12 (0.80-1.57) | 1.24 (0.94-1.62) | 1.11 (0.79-1.55) | 1.08 (0.82-1.43) |

CI: Confidence Interval; HR: Hazard Ratio; SES: Socioeconomic status

^a^ Adjusted for age, sex, ethnicity, family history of diabetes, prevalent conditions.

^b^ The cumulative SES score is entered as a continuous 3-level variable into the models. Hazard ratio is for the lowest vs. highest score.

^c^ Lifestyle factors considered are smoking, diet, physical activity, body mass index, inflammatory markers.
