# Supplementary material for: Association of Lifecourse Socioeconomic Status with Chronic Inflammation and Type 2 Diabetes Risk: The Whitehall II Prospective Cohort Study
Source: PLoS Med. 2013 Jul 2;10(7):e1001479. doi: 10.1371/journal.pmed.1001479 (PMC3699448; doi:10.1371/journal.pmed.1001479)
Supplement: Table S6 — Association of cumulative socioeconomic score with type 2 diabetes incidence ( n = 6,198; 641 incident diabetes cases). Additional adjustment for pack years of cigarettes smoked, drug intake, alcohol consumption, height, and BMI at 25 y. (DOCX) [file pmed.1001479.s007.docx]

**Table S6. Association of cumulative socioeconomic score with type 2 diabetes incidence (N=6198; 641 incident diabetes cases) ADDITIONAL ADJUSTMENT FOR PACK YEARS OF CIGARETTES SMOKED, DRUG INTAKE, ALCOHOL CONSUMPTION, HEIGHT AND BMI AT 25 years.**

| **Cumulative SES score^a^** | **HR (95%CI)** | **%Δ** |
| --- | --- | --- |
| **Model 1:** Adjusted for age, sex, ethnicity family history and prevalent conditions | 2.10 (1.56-2.82) | Ref. |
| **Model 2:** Model 1 + smoking status^b^ | 2.03 (1.51-2.73) | -5 |
| **Model 3:** Model 1 + pack years^b^ | 2.08 (1.54-2.80) | -1 |
| **Model 4:** Model 1 + physical activity^b^ | 2.06 (1.53-2.77) | -3 |
| **Model 5:** Model 1 + diet^b^ | 2.02 (1.50-2.72) | -5 |
| **Model 6:** Model 1 + drinking^b^ | 2.09 (1.55-2.81) | -1 |
| **Model 7:** Model 1 + BMI^b^ | 1.84 (1.36-2.48) | -18 |
| **Model 8:** Model 1 + height and BMI at 25y^b^ | 1.95 (1.544-2.62) | -10 |
| **Model 9:** Model 1 + drug intake^b^ | 2.06 (1.53-2.78) | -2 |
| **Model 10:** Model 1 + smoking status, pack years cigarettes smoked, physical activity, diet, BMI, height, BMI at 25, drug intake^b^ | 1.68 (1.32-2.12) | -30 |
| **Model 11:** Model 1 + CRP^b^ | 1.84 (1.37-2.47) | -18 |
| **Model 12:** Model 1 + IL-6^b^ | 1.91 (1.42-2.57) | -13 |
| **Model 13:** Model 1 + CRP +IL-6^b^ | 1.79 (1.33-2.40) | -22 |
| **Model 14:** Model 1 + all risk factors^b^ | 1.62 (1.19-2.19) | -35 |
| Additional contribution of CRP+IL-6 to Model 5^b^ |  | **-7^c^** |

BMI: Body Mass Index; CI: Confidence Interval; CRP: C - reactive protein; HR: Hazard Ratio; IL-6: Interleukin-6; Ref: Reference; SES: Socioeconomic Status; Δ: Attenuation

^a^ The cumulative SES score is entered as a continuous 3-level variable into the models. Hazard ratio is for the lowest vs. highest score.

^b^ All risk factors are updated at Phases 3, 5 &7 and additionally adjusted for the risk factor at the previous phase.

^c^Additional contribution of CRP and IL-6 to the model adjusted for age, sex, ethnicity, family history of diabetes, prevalent conditions, smoking, physical activity, alcohol consumption, diet, BMI, antihypertensive and lipid lowering drugs.
