# Supplementary material for: Association of Lifecourse Socioeconomic Status with Chronic Inflammation and Type 2 Diabetes Risk: The Whitehall II Prospective Cohort Study
Source: PLoS Med. 2013 Jul 2;10(7):e1001479. doi: 10.1371/journal.pmed.1001479 (PMC3699448; doi:10.1371/journal.pmed.1001479)
Supplement: Table S7 — Association of cumulative socioeconomic score with type 2 diabetes incidence ( n = 6,387; 731 incident diabetes cases). Age as the time scale. (DOCX) [file pmed.1001479.s008.docx]

**Table S7. Association of cumulative socioeconomic score with type 2 diabetes incidence (N=6387; 731 incident diabetes cases) AGE AS THE TIME SCALE**

| **Cumulative SES score^a^** | **HR (95%CI)** | **%Δ** |
| --- | --- | --- |
| **Model 1:** Adjusted for year of birth, sex, ethnicity family history and prevalent conditions | 1.95 (1.48-2.58) | Ref. |
| **Model 2:** Model 1 + smoking^b^ | 1.87 (1.42-2.48) | -6 |
| **Model 3:** Model 1 + physical activity^b^ | 1.90 (1.44-2.52) | -4 |
| **Model 4:** Model 1 + diet^b^ | 1.85 (1.40-2.45) | -8 |
| **Model 5:** Model 1 + BMI^b^ | 1.71 (1.29-2.27) | -20 |
| **Model 6:** Model 1 + smoking, physical activity, diet and BMI^b^ | 1.57 (1.18-2.09) | -30 |
| **Model 7:** Model 1 + CRP^b^ | 1.69 (1.28-2.23) | -22 |
| **Model 8:** Model 1 + IL-6^b^ | 1.79 (1.35-2.36) | -13 |
| **Model 9:** Model 1 + CRP +IL-6^b^ | 1.65 (1.24-2.18) | -25 |
| **Model 10:** Model 1 + all risk factors^b^ | 1.50 (1.13-2.00) | -39 |
| Additional contribution of CRP+IL-6 to Model 5^b^ |  | **-12^c^** |

BMI: Body Mass Index; CI: Confidence Interval; CRP: C - reactive protein; HR: Hazard Ratio; IL-6: Interleukin-6; Ref: Reference; SES: Socioeconomic Status; Δ: Attenuation

^a^ The cumulative SES score is entered as a continuous 3-level variable into the models. Hazard ratio is for the lowest vs. highest score.

^b^ All risk factors are updated at Phases 3, 5 &7 and additionally adjusted for the risk factor at the previous phase.

^c^Additional contribution of CRP and IL-6 to the model adjusted for year of birth, sex, ethnicity, family history of diabetes, prevalent conditions, smoking, physical activity, BMI and diet.
