# Supplementary material for: Association of Lifecourse Socioeconomic Status with Chronic Inflammation and Type 2 Diabetes Risk: The Whitehall II Prospective Cohort Study
Source: PLoS Med. 2013 Jul 2;10(7):e1001479. doi: 10.1371/journal.pmed.1001479 (PMC3699448; doi:10.1371/journal.pmed.1001479)
Supplement: Table S8 — Association of cumulative socioeconomic score with type 2 diabetes incidence ( n = 8,526; 909 incident diabetes cases). Multiple imputation (STATA ICE/micombine procedures). (DOCX) [file pmed.1001479.s009.docx]

**Table S8. Association of cumulative socioeconomic score with type 2 diabetes incidence (N=8526; 909 incident diabetes cases) MULTIPLE IMPUTATION (STATA ICE/MICOMBINE PROCEDURES)**

| **Cumulative SES score^a^** | **HR (95%CI)** | **%Δ** |
| --- | --- | --- |
| **Model 1:** Adjusted for year of birth, sex, ethnicity family history and prevalent conditions | 1.61 (1.25-2.09) | Ref. |
| **Model 2:** Model 1 + smoking^b^ | 1.56 (1.20-2.02) | -7 |
| **Model 3:** Model 1 + physical activity^b^ | 1.56 (1.21-2.02) | -6 |
| **Model 4:** Model 1 + diet^b^ | 1.54 (1.18-1.99) | -10 |
| **Model 5:** Model 1 + BMI^b^ | 1.37 (1.05-1.78) | -34 |
| **Model 6:** Model 1 + smoking, physical activity, diet and BMI^b^ | 1.27 (0.97-1.65) | -50 |
| **Model 7:** Model 1 + CRP^b^ | 1.46 (1.13-1.89) | -21 |
| **Model 8:** Model 1 + IL-6^b^ | 1.51 (1.16-1.96) | -14 |
| **Model 9:** Model 1 + CRP +IL-6^b^ | 1.43 (1.11-1.87) | -24 |
| **Model 10:** Model 1 + all risk factors^b^ | 1.21 (0.93-1.68) | -56 |
| Additional contribution of CRP+IL-6 to Model 5^b^ |  | **-11^c^** |

BMI: Body Mass Index; CI: Confidence Interval; CRP: C - reactive protein; HR: Hazard Ratio; IL-6: Interleukin-6; Ref: Reference; SES: Socioeconomic Status; Δ: Attenuation

^a^ The cumulative SES score is entered as a continuous 3-level variable into the models. Hazard ratio is for the lowest vs. highest score.

^b^ All risk factors are updated at Phases 3, 5 &7 and additionally adjusted for the risk factor at the previous phase.

^c^Additional contribution of CRP and IL-6 to the model adjusted for year of birth, sex, ethnicity, family history of diabetes, prevalent conditions, smoking, physical activity, BMI and diet.
